# Pharmacologic Inhibition of S-Nitrosoglutathione Reductase Prevents Hyperoxic Alveolar and Airway Disease in Newborn Mice

**DOI:** 10.3390/biomedicines14010015

**Published:** 2025-12-20

**Authors:** Stephanie Adaikalam, Ramadan B. Sopi, Laura A. Smith, Anjum Jafri, Peter M. MacFarlane, Richard J. Martin, Benjamin Gaston, Thomas M. Raffay

**Affiliations:** 1 Division of Pediatric Pulmonology, Allergy, and Sleep Medicine, Department of Pediatrics, Indiana University, Indianapolis, IN 46202, USA; sadaikal@iu.edu (S.A.); las13@iu.edu (L.A.S.); begaston@iu.edu (B.G.); 2 School of Medicine, Case Western Reserve University, Cleveland, OH 44106, USA; rxs274@case.edu (R.B.S.); axj21@case.edu (A.J.); pmm71@case.edu (P.M.M.); richard.martin@uhhospitals.org (R.J.M.); 3 Faculty of Medicine Department of Premedical Courses, University of Prishtina, 10000 Prishtina, Kosovo; 4 Division of Neonatal and Perinatal Medicine, Department of Pediatrics, UH Rainbow Babies & Children’s Hospital, Euclid Avenue, Cleveland, OH 44106, USA

**Keywords:** bronchopulmonary dysplasia, airway hyperreactivity, oxygen, S-nitrosothiol

## Abstract

**Background/Objectives**: Preterm infants are at risk of developing the chronic lung condition of bronchopulmonary dysplasia (BPD), with associated alveolar simplification and airway hyperreactivity. Inhibition of S-nitrosoglutathione (GSNO) reductase has been shown to rescue airway hyperreactivity in a murine model of BPD. Here, we investigate the effects of early treatment with N6022, a pharmacologic GSNO reductase inhibitor. **Methods:** Newborn C57BL/6 mice were exposed to either 21% (control) or 60% oxygen (BPD model) for 5 days after birth. Pups simultaneously received either subcutaneous saline or varying doses of N6022 for 5 days during hyperoxia exposure. Pups were then recovered in room air to 3 weeks postnatal age. H&E-stained lungs were analyzed for alveolar simplification and airway tethering. In vivo airway reactivity to inhaled methacholine was measured using a flexiVent system. In separate littermates, lungs were immediately harvested after 5 days of hyperoxia for protein quantification via automated capillary Westerns. **Results:** Alveolar simplification and decreased airway tethering were noted in the 60% + saline group. Pups treated with N6022 during hyperoxia displayed dose-dependent improvements in alveolar simplification and airway tethering. Similarly, hyperoxia-exposed pups had increased airway reactivity, as measured by elevated respiratory system resistance and elastance responses to methacholine. Treatment with 10 mg/kg/day N6022 during hyperoxia resulted in decreased resistance and elastance responses. TGF-β expressions were elevated in the 60% + saline group and attenuated in the 60% + N6022 groups. **Conclusions:** Early exposure to GSNO reductase inhibitors such as N6022 can prevent hyperoxia-induced alveolar simplification and airway hyperreactivity, with lasting effects even after cessation of treatment.

## 1. Introduction

Approximately 10% of infants are born prematurely [1,2] and complications from preterm birth are the leading cause of death in children under 5 years of age [3]. Infants born prematurely are at increased risk of lung disease, such as bronchopulmonary dysplasia (BPD), which is defined by premature birth prior to 32 weeks gestational age and continued need for respiratory support at 36 weeks postmenstrual age [4]. Even in infants born prematurely who do not meet the definition of BPD, many patients go on to have childhood respiratory symptoms and obstruction on pulmonary function tests into adulthood [5,6].

Lung disease of prematurity is multi-factorial, resulting from interrupted development and exposure to a high inflammatory state, as well as iatrogenic exposures to hyperoxia and positive pressure ventilation. The lungs of infants born prematurely in the post-surfactant era demonstrate alveolar simplification and disruptions in angiogenesis [7]. Clinically, BPD results in bronchial hyperreactivity, as well as the risk of other phenotypes including tracheobronchomalacia, pulmonary hypertension, and lymphatic dysfunction [8]. Despite the numerous comorbidities of BPD, there are few FDA-approved drugs in neonates (i.e., caffeine, steroids, vitamin A) [9] that have shown efficacy in randomized controlled trials. Novel therapeutic pathways are needed to treat and prevent the sequelae of BPD.

S-Nitrosothiols have emerged as a potential target for both the treatment and prevention of BPD. S-Nitrosothiols are signaling molecules that regulate the biologic activity of proteins post-translationally via S-nitrosylation. One such S-nitrosothiol is endogenously formed from the antioxidant glutathione to form S-nitrosoglutathione (GSNO). GSNO is a potent bronchodilator [10] that decreases inflammation [11]. While there are numerous proteins relevant in GSNO metabolism, GSNO reductase encoded by the *ADH5* gene is its primary catabolic enzyme [12]. GSNO reductase is known to be increased in patients with asthma, resulting in increased bronchoreactivity [13]. In mouse models, GSNO reductase knockout protects against asthma [14] and beta-adrenergic receptor tachyphylaxis [15], as well as myocardial injury [16]. Similarly, autopsied lungs from infants with BPD also showed increased GSNO reductase expression compared to those without BPD [17].

Mice are born with immature, saccular lungs in a similar stage of lung development as infants born at 26–28 weeks gestational age [18]. Newborn hyperoxia mouse models demonstrate a phenotype similar to BPD, including alveolar simplification, airway hyperreactivity, and pulmonary hypertension [19,20]. Our work and others have previously shown increased GSNO catabolism and/or increased GSNO reductase expression in hyperoxia BPD models compared to room air controls [21,22]. In addition to developing airway hyperreactivity, we have shown that newborn hyperoxia decreases potentially beneficial GSNO bronchodilatory effects in the intrapulmonary bronchioles of adult mice [23]. Acute pharmacologic GSNO reductase inhibition restores this effect to match room air controls [21,23], demonstrating that GSNO reductase inhibition improves bronchial hyperreactivity and restores GSNO bronchodilation seen in this BPD model. In addition to these treatment effects, we have also demonstrated that GSNO reductase gene deletion prevents pathologic changes seen in the murine BPD model [17]. While wild-type mice exposed to hyperoxia have decreased alveolar counts, increased airway hyperreactivity, increased right ventricular mass, and increased arterial wall thickness, the GSNO reductase global knockout model is undifferentiated from the room air wild- type by each of these characteristics. Further work is needed to evaluate these preventative effects using chemical GSNO reductase inhibition in vivo. The current work aims to evaluate the efficacy of a GSNO reductase inhibitor that was well-tolerated in human asthma clinical trials [24], as a preventive therapy of murine BPD lung and airway disease when given during newborn oxygen exposure.

## 2. Materials and Methods

Animals: Timed-pregnant C57BL/6 mice were purchased from a commercial vendor (Charles River Laboratories, Wilmington, MA, USA). Within 24 h of birth, two or more litters of mixed male and female pups were pooled and then randomized to treatment and exposure groups beginning on postnatal day one (P1). Randomized pups were paired with a nursing female and cages were kept on 12 h light–dark cycles with ad libitum standard food and water in the same room. Each experimental outcome was comprised of two or more pooled litters consisting of offspring from four or more pregnant females; n represents individual animals. All procedures were carried out in accordance with the National Institutes of Health guidelines for care and use of laboratory animals and were approved by the Case Western Reserve University Institutional Animal Care and Use Committee (Assurance Number A-3145-01).

**Figure 1:**
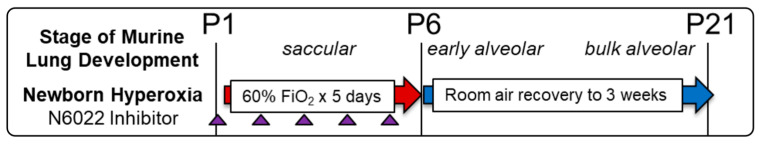
Experimental Model. Newborn mice were treated daily with subcutaneous injections of saline or the GSNO reductase inhibitor, N6022, on postnatal days 1–5 (purple triangles). Mice were exposed to room air (21% oxygen) or hyperoxia (60% oxygen) on postnatal days 1–6 during the murine saccular stage of lung development. Mice were either immediately collected on P6 or recovered in room air to 3 weeks of age during the murine early and bulk alveolar stages of lung development. Created in BioRender. Adaikalam, S. (2025) https://BioRender.com/h30kls1.

Neonatal Inhibitor Treatment and Oxygen Exposures: Pups received daily (P1-5) subcutaneous injections of saline (0.9% sodium chloride, Baxter Healthcare, Deerfield, IL, USA) or the GSNO reductase inhibitor N6022 (0.1, 1, or 10 mg/kg, CAS: 1208315-24-5, Millipore Sigma, Milwaukee, WI, USA) during the 5 days of consecutive exposures to room air (21% oxygen) or hyperoxia (60% oxygen) (Figure 1). The small molecule reversible GSNO reductase inhibitor (N6022) [25] doses were selected based on previously reported murine dose ranges and toxicology studies [19,26]. Animals raised in hyperoxia were housed in standard cages placed in a 38-L Plexiglas chamber with a continuous flow of blended oxygen (2–3 L/min) with a contra-lateral ventilation port to equilibrate chambers to atmospheric pressure. Animals and oxygen concentrations were monitored daily via an oxygen analyzer (miniOX I; Ohio Medical, Gurnee, IL, USA). In a subset of pups from each group, tissues were harvested immediately after 5 days of exposure (P6) for protein assays. The remaining littermate pups from each group were returned to room air until 3 weeks of age (P21) for lung morphology or respiratory mechanics assessments.

Lung Micrographs: After terminal anesthesia (ketamine/xylazine; Pfizer, St Joseph, MO, USA and Lloyd Laboratories, Shenandoah, IA, USA), lungs of P21 mice were inflation-fixed with intratracheal 4% paraformaldehyde (Cat #15714-S, Electron Microscopy Sciences, Hatfield, PA, USA) at a sustained pressure of 25 cm H_2_O, the trachea was tied-off, and the lung and trachea were post-fixed en bloc in 4% paraformaldehyde for >24 h at 4 °C and then dehydrated in 70% alcohol. The left lung was excised, embedded in paraffin, and sectioned at 5 μm. Mounted slides were de-paraffinized using xylene and then rehydrated in graded alcohol. Hematoxylin and eosin staining (H&E) was performed for measurements of mean linear intercepts (MLI) and bronchoalveolar attachments under light microscopy (Leica Microsystems, Wetzlar, Germany) with a mounted digital camera (QImaging Retiga EXi 12-bit camera, Surrey, BC, Canada) [27]. Nine or more non-overlapping images from 3 lung sections per animal were analyzed with ImageJ software (Image J 1.52v, NIH, Bethesda, MD, USA) utilizing the “Measure MLI” plug-in [28,29] using a 150-pixel reference. Bronchoalveolar attachments were measured from a minimum of 10 airways per animal and reported as the number of attachments divided by the airway perimeter [30].

Respiratory Mechanics: Using a rodent ventilator (flexiVent FX-1, SCIREQ, Montreal, QC, Canada), in vivo respiratory system mechanics were measured in response to increasing doses of aerosolized methacholine in 3-week-old mice as previously described [21]. Under anesthesia (intraperitoneal ketamine/xylazine), mice were placed supine on a heated surgical table, tracheostomized, and ventilated via a sutured 19G blunt tip cannula at default settings: tidal volume of 10 mL/kg, a rate of 150 breaths/min, a PEEP of 3 cm H_2_O, and FiO_2_ of 50%. A muscle relaxant was given to prevent spontaneous breathing (intraperitoneal pancuronium bromide, Cat #P1918, Sigma-Aldrich, St. Louis, MO, USA). Following two recruitment breaths of sustained inflation at a pressure of 30 cm H_2_O for 3 s, saline vehicle (Cat #14190144, Thermo Fisher, Waltham, MA, USA) was aerosolized over 10 s using an ultrasonic nebulizer (Aeroneb; SCIREQ, Montreal, QC, Canada) diverted into the ventilator’s inspiratory flow. After five minutes had elapsed, two recruitment breaths were again delivered and increasing methacholine doses of 6, 12.5, 25, 50, 100, and 200 mg/mL in saline were similarly aerosolized over 10 s to generate a dose-response curve (Cat #A2251, Sigma-Aldrich, St. Louis, MO, USA). Using computer software (flexiWare 5.1, SCIREQ, Montreal, QC, Canada), replicate measurements of respiratory system resistance (Rrs) and elastance (Ers) were calculated by a 2.5 Hz single-frequency forced oscillation maneuver (Snapshot 150) fit to the linear equation of motion using a single compartment model [20]. Animals were continuously monitored by electrocardiogram and transduced airway peak inspiratory pressure during mechanical ventilation. All animals analyzed completed the full methacholine dose response. Replicates were averaged for each methacholine dose in each individual animal.

**Figure A1:**
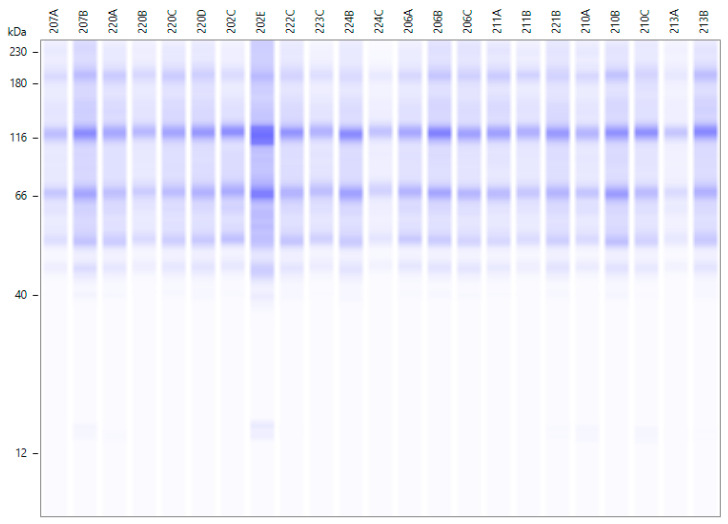
Representative Simple Western Total Protein measurement from the TGF-β run. Chemiluminescent detection was performed for each target protein using the Simple Western platform (ProteinSimple, Bio-Techne). The same capillaries were reprobed using the RePlex total protein assay for normalization in order to account for variability due to sample loading. The figure is a representative example of the total protein measurements, which were performed for each assay.

Automated Capillary Western Blot System: After terminal anesthesia (ketamine/xylazine) lungs of P6 mice were harvested, rinsed in ice-cold phosphate-buffered saline (PBS, pH 7.4), snap-frozen, and stored at −80 °C. To prepare lung lysate for protein analysis, a small piece of mouse lung was excised. Tissue was lysed in radioimmunoprecipitation assay (RIPA) buffer (Cat #R0278, Sigma-Aldrich, Saint Louis, MO, USA) with protease inhibitor (Cat #539134, Millipore Sigma, Milwaukee, WI, USA) using mechanical homogenization. Lysates were centrifuged at maximum speed (16,000× *g*) for 10 min at 4 °C, and the supernatant was collected. Total protein concentration of the clarified lung lysate was measured using the Pierce bicinchoninic acid (BCA) assay (ThermoFisher Scientific, Waltham, MA, USA) according to the manufacturer’s instructions. Samples were diluted in 0.1× sample buffer (Cat #042-195, ProteinSimple, San Jose, CA, USA) to a final concentration of 1.5 µg/µL of protein. Target proteins were quantified in each lysate using the Automated Western Blot System (ProteinSimple). Primary antibodies included TGF-β, IL-1β, and nitrotyrosine. The optimal loading sample protein concentration, antibody dilution factor, and antibody diluent (D2 or milk-free buffer) were determined empirically for each antibody (Table A1). Each lung lysate was further diluted in 0.1× sample buffer to the optimized concentration for that antibody before being loaded into the Simple Western Automated Western Blot System. Compass software (version 6.3.0, ProteinSimple) was used to measure peaks near the expected molecular weight for each antibody. For nitrotyrosine, all quantified peaks were summed to obtain the total nitrotyrosine signal. Total protein measurements were used as the loading control for normalization for all proteins of interest, according to the manufacturer’s Replex workflow (ProteinSimple, San Jose, CA, USA) (Figure A1). This method allows the total protein signal to be measured within the same capillary as the target protein, allowing for accurate normalization and eliminating the need for housekeeping proteins that can vary by disease state and treatment [31]. The ratio of the antibody of interest to total protein was used in all analyses.

Statistical Analysis: Results are expressed as means (±SEM). The Shapiro–Wilk test was used to assess for normality. When data passed normality testing, results were analyzed by analysis of variance (ANOVA) with post hoc comparisons as indicated for multiple groups using statistical software (SigmaPlot Version 12.0; Systat Software, San Jose, CA, USA). When the normality test failed, Kruskal–Wallis One-Way Analysis of Variance on Ranks was used to assess for group differences, followed by pairwise multiple comparison via Dunn’s Method. Methacholine dose-responses were analyzed via two-way repeated measures ANOVA by group and dose with stepwise post hoc comparisons starting at the maximal response and moving to lower methacholine doses until significance was no longer detected [20]. Secondary analyses of flexiVent data for an interaction between animal sex, exposure group, and dose were performed using a general linear model with the dependent variables of Rrs and Ers, respectively. A significance threshold of *p* < 0.05 was used for all analyses.

## 3. Results

### 3.1. Evaluation of Alveolar Differences on H&E Stain at P21

#### 3.1.1. Measurements of Alveolar Simplification

**Figure 2:**
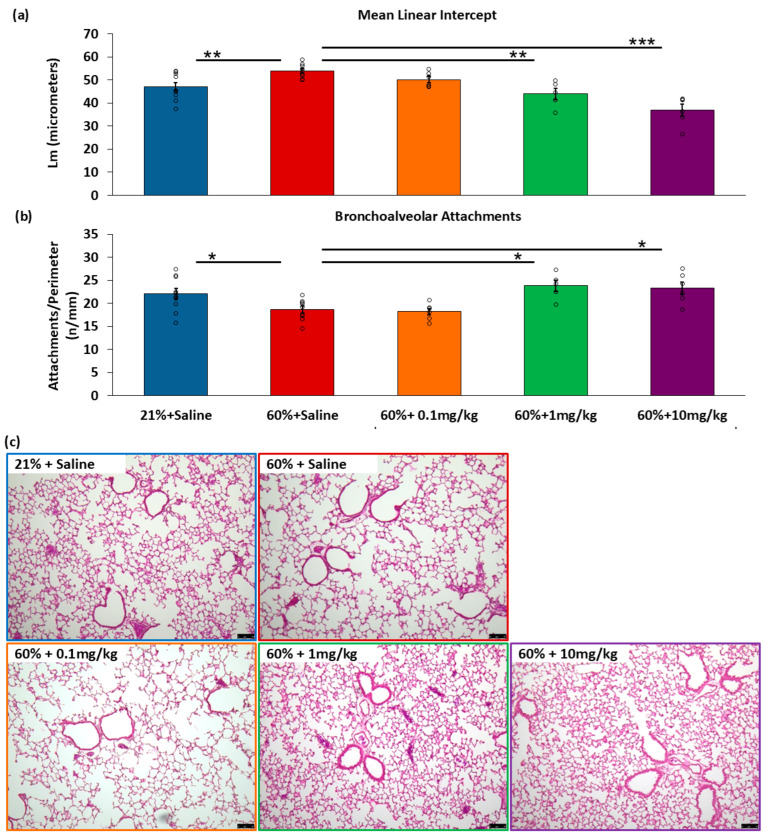
Daily GSNO reductase inhibitor protects against hyperoxia-induced alveolar simplification. H&E stains of lungs harvested at P21 were evaluated for mean linear intercept and bronchoalveolar attachments/perimeter. In both evaluations, the 60% + saline group was significantly different from the 21% + saline (room air controls). The 60% + 0.1 mg/kg N6022 group did not significantly differ from the 60% + saline group, but the 1 mg/kg and 10 mg/kg doses in hyperoxia showed preserved alveolarization by (**a**) L_m_ and increased airway tethering by attachments/perimeter (**b**). Representative lung images (**c**), scale bar = 75 µm. Means ± SEM. Individual data points are represented with circles. One-way ANOVA with post hoc Holm–Šídák comparisons. * *p* < 0.05, ** *p* < 0.01, *** *p* < 0.001 vs. 60% + saline.

After recovery in room air and terminal anesthesia at P21, inflation-fixed lung sections were stained with H&E and analyzed. Mean Linear Intercepts (L_m_) were evaluated, where increased L_m_ represents increased space between alveoli and alveolar simplification. The 60% + saline mice (*n* = 9) had higher L_m_ compared to room air controls treated with saline (*n* = 10). The mice treated with a GSNO reductase inhibitor, N6022, exhibited a dose-dependent decrease in L_m_. The mice exposed to 60% oxygen and given the lowest dose of N6022 (0.1 mg/kg, *n* = 6) showed no significant decrease in L_m_ compared to the 60% + saline group (*p* = 0.15). In contrast, the mice exposed to 60% oxygen and given 1 mg/kg (*n* = 5) or 10 mg/kg N6022 (*n* = 6) were protected from alveolar simplification as measured by L_m_ (Figure 2a).

#### 3.1.2. Measurements of Airway Tethering

Similarly, the number of bronchoalveolar attachments was also assessed in the H&E-stained lung slices as a measure of airway tethering. The mice exposed to 60% oxygen treated with saline had decreased attachments/perimeter compared to the room air controls, indicating reduced airway tethering. The hyperoxia-exposed mice given 0.1 mg/kg N6022 did not differ from the 60% + saline group, but the mice given 1 mg/kg or 10 mg/kg N6022 had increased attachments/perimeter compared to 60% + saline (Figure 2b).

### 3.2. Measurements of In Vivo Respiratory System Reactivity at P21

**Figure 3:**
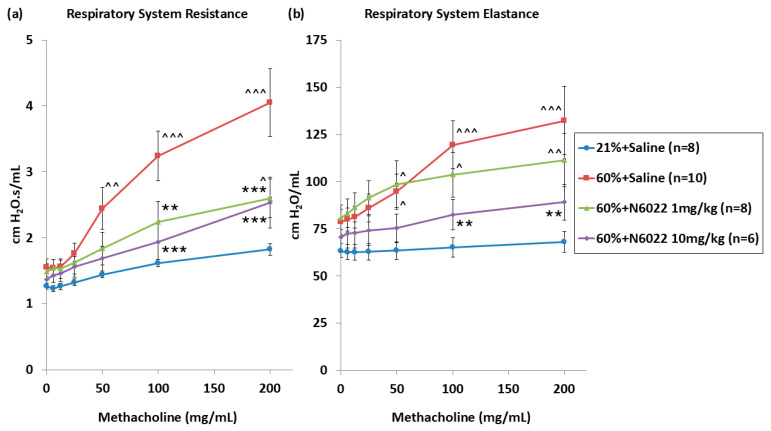
Daily GSNO reductase inhibitor protects against hyperoxia-induced airway hyperreactivity. Respiratory system resistance (**a**) and elastance (**b**) were measured in vivo at 3 weeks. Hyperoxia-exposed mice (60% + saline, *n* = 10, red closed-square) demonstrated increased airway resistance and elastance compared to room air control mice (21% + saline, *n* = 8, blue closed-circle). Hyperoxia-exposed mice treated with the GSNO reductase inhibitor N6022 (60% + 1 mg/kg, *n* = 8, green closed-triangle and 60% + 10 mg/kg, *n* = 6, purple closed-diamond) had decreased respiratory system resistance responses compared to the 60% + saline group. Hyperoxia-exposed mice treated with 10 mg/kg N6022 had decreased respiratory system elastance responses compared to the 60% + saline group. Means ± SEM. Individual data points are provided in the Supplementary Material (Table S1). Two-way repeated-measures ANOVA with post hoc Holm–Šídák comparisons by group and dose. ** *p* < 0.01, *** *p* < 0.001 vs. 60% + saline. ^ *p* < 0.05, ^^ *p* < 0.01, ^^^ *p* < 0.001 vs. 21% + Saline.

Respiratory system resistance (Rrs) and elastance (Ers) were measured with increasing doses of inhaled methacholine in three-week-old mice to test the effects of daily 1 or 10 mg/kg N6022 dosing during hyperoxia. The hyperoxia-exposed mice (*n* = 10) showed significantly higher Rrs and Ers compared to the room air control mice (*n* = 8) at inhaled methacholine doses of 50, 100, and 200 mg/mL, representing increased airway hyperreactivity in this hyperoxic BPD model (Figure 3a,b). The hyperoxia-exposed mice that were earlier treated with N6022 at 1 mg/kg (*n* = 8) or 10 mg/kg (*n* = 6) had reduced Rrs compared to the hyperoxia + saline mice at the inhaled methacholine doses of 100 and 200 mg/mL. Compared to room air controls, Rrs and Ers responses in the 10 mg/kg N6022 group did not significantly differ at any methacholine dose. In the 1 mg/kg N6022 group, Rrs responses were significantly different from those of room air controls at a methacholine dose of 200 mg/mL (*p* = 0.039 vs. 21% + Saline). Ers responses in the 1 mg/kg N6022 group were significantly different from those of room air controls at methacholine doses of 50, 100, and 200 mg/mL. This demonstrated that pharmacologic inhibition of GSNO reductase during neonatal hyperoxia protected mice against later hyperreactivity measured as increased resistance. Using a general linear model, a significant interaction was not detected between animal sex with exposure group and methacholine dose.

### 3.3. Evaluation of Lung Proteins Following Newborn Hyperoxia at P6

**Figure 4:**
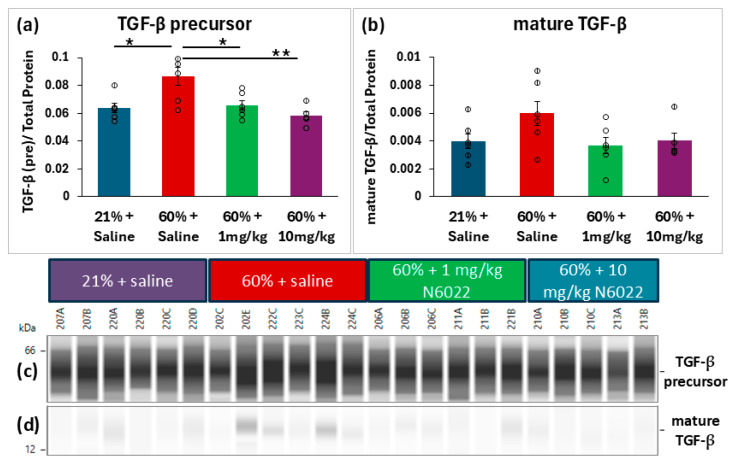
TGF-β is decreased in hyperoxia-exposed mice simultaneously treated with N6022. Chemiluminescence peaks from capillary-based Simple Western blots were used to quantify TGF-β at molecular weights of (**a**) 59k Daltons (precursor) and (**b**) 17k Daltons (mature). The results are visualized as an automated lane view, representing the intensity of the chemiluminescence peaks for the precursor (**c**) and mature (**d**) TGF-β proteins. A representative image of the total protein measurements used for normalization can be found in Figure A1. Chemiluminescence peaks and full uncropped images can be found in Figure S1a,b. The saline-treated hyperoxia-exposed mice demonstrated elevated expression of precursor TGF-β. Mice treated with N6022 did not significantly differ from room-air control mice. Means ± SEM. Individual data points are represented with circles. One-way ANOVA with post hoc Holm–Šídák comparisons. * *p* < 0.05, ** *p* < 0.01 vs. 60% + saline.

**Figure 5:**
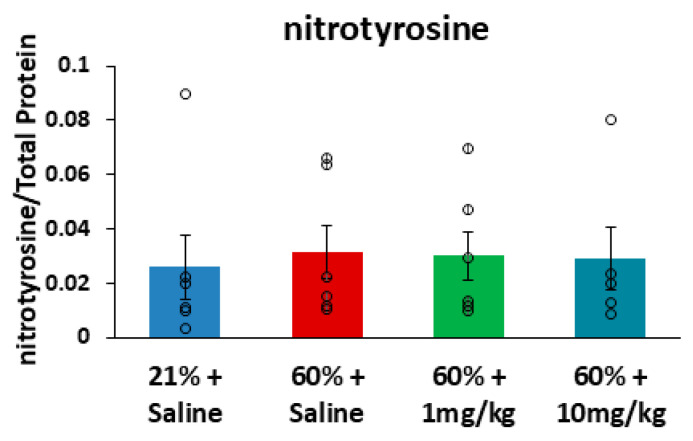
No difference detected in nitrotyrosine end-products. The sum of all nitrotyrosine peaks was used to quantify nitrotyrosine end-products. Total nitrotyrosine burden was compared across the groups with no statistical differences, demonstrating no increased protein damage with N6022 treatment. The results are visualized as an automated lane view, representing the intensity of all chemiluminescence peaks (Figure S2a,b). Means ± SEM. Individual data points are represented with circles. Kruskal–Wallis One-Way Analysis of Variance on Ranks was performed (*p* = 0.889). Post hoc pairwise comparison was not performed since the ANOVA did not detect significant differences.

**Figure A2:**
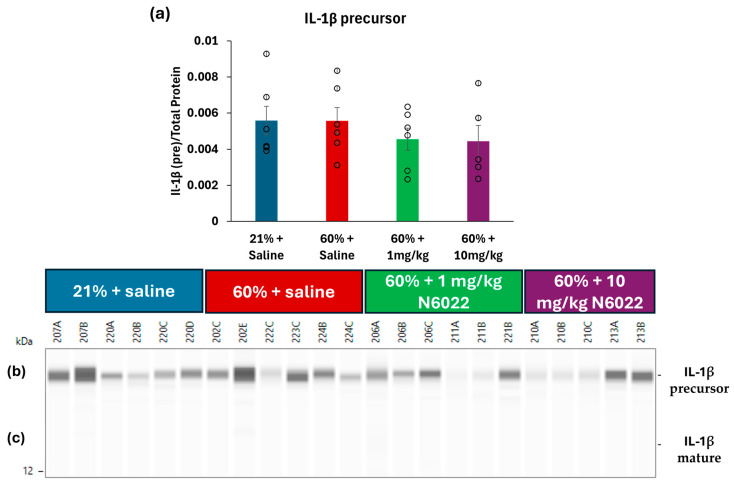
No significant differences among experimental groups in IL-1β precursor expression (~32k Daltons, *p* = 0.64). (**a**) Quantified IL-1β precursor to total protein ratio shows no difference between experimental groups, visually represented by the lane view (**b**). Mature IL-1β (~17k Daltons) was not reliably detected by the lane view (**c**). In all samples, the signal intensities at this molecular weight were near background levels and below acceptable signal-to-noise thresholds for quantification. Means ± SEM. Individual data points are represented with circles. One-way ANOVA was performed (*p* = 0.643). Post hoc comparisons were not performed since no significant differences were detected. Full uncropped images can be found in Figure S3.

Immediately after newborn hyperoxia exposures, whole lungs from P6 mice were snap-frozen for analyses of inflammatory proteins to test the effects of daily 1 or 10 mg/kg N6022 dosing during hyperoxia. Automated capillary Simple Westerns were performed on lung homogenates to compare relative expressions of TGF-β, IL-1β, and nitrotyrosine. Expression of the TGF-β precursor protein was found to be elevated in the hyperoxia-exposed mice (*n* = 6) compared to the room air controls (*n* = 6) (*p* = 0.013), while treatment with N6022 at either 1 mg/kg (*n* = 6) or 10 mg/kg (*n* = 5) during hyperoxia reduced expression of the TGF-β precursor to levels that did not significantly differ from room air controls (Figure 4a). A similar pattern was observed for expression of mature TGF-β; however, one-way ANOVA did not detect a significant effect (*p* = 0.11); thus, post hoc testing was not performed to compare groups (Figure 4b). Another inflammatory marker, IL-1β, was also evaluated with no significant group differences detected (Figure A2). The sum of all nitrotyrosine peaks was measured as an assessment of protein damage in the form of nitrotyrosine end-products. There were no differences in total nitrotyrosine signals across the four groups (*p* = 0.889) (Figure 5). Chemiluminescence peaks and uncropped lane views from the capillary-based automated Westerns can be found in Supplementary Materials (Figures S1–S3).

### 3.4. Assessment for N6022-Associated Mortality and Morbidity

Treatment with N6022 appeared to be well-tolerated at all three tested doses. There was no mortality in any of the N6022 inhibitor groups during daily treatments. Animals were observed daily by facility staff to have normal development of ambulation, feeding, and grooming behaviors to the age of P21. There were no significant differences detected in P5 or P21 bodyweight between groups (Table A2).

## 4. Discussion

Current treatment of respiratory disease in premature infants with BPD is primarily limited to corticosteroids and beta-agonists, drugs which have been used in this setting since the 1980s [32,33]. Minimal progress has been made in therapeutic options for this population, and there are currently no approved therapies indicated for the treatment or prevention of BPD in infants born prematurely. Disease-specific drug targets are greatly needed.

Prior work has demonstrated that GSNO reductase is elevated in the lungs of infants with BPD [17]. Hyperoxia rodent models have similarly reported increased lung GSNO reductase [21,22], potentially through oxygen-induced dysregulated micro-RNA-342 expression [21,34,35]. In a moderate hyperoxia model of BPD, inhibiting GSNO reductase after hyperoxia exposure has been shown to rescue airway hyperreactivity and restore bronchodilation [21,23]. In addition, we have previously shown that GSNO reductase genetic knockout mice were protected from developing lung and airway disease secondary to hyperoxia [17]. Similarly, alveolar protection with N6022 has been reported in continuous newborn hyperoxia [22] and in an adult emphysema model [36]. In a hyperoxia/hypoxia neonatal rat model, administration of sodium nitrite increased lung S-nitrosothiols, providing alveolar protection; notably, in this model inhaled nitric oxide did not effectively increase lung S-nitrosothiols nor provide alveolar protection [37]. Prior to the current study, the effects of chemical inhibition of GSNO reductase during neonatal hyperoxia exposure on lung and airway disease had not been evaluated.

In the current study, newborn mice were given varying doses of the GSNO reductase inhibitor, N6022, during the initial exposure to hyperoxia. GSNO reductase inhibitors have been tested using similar dosage regimens in pre-clinical pulmonary studies, including rodent models of asthma, emphysema, and severe acute respiratory syndrome coronavirus-2 (SARS-CoV-2) [36,38,39]. We re-demonstrated that hyperoxia-exposed mice develop alveolar simplification, decreased airway tethering, and increased airway hyperreactivity compared to room air controls. In mice simultaneously treated with a GSNO reductase inhibitor during hyperoxia, these effects are diminished in a dose-dependent manner. At a dose of 1 mg/kg, N6022 given during hyperoxia exposure improved outcomes measured weeks later. This included protections from alveolar simplification and improved airway tethering, as well as significantly reduced Rrs responses to induced bronchoconstriction. At a dose of 10 mg/kg, the effects of N6022 given during hyperoxia exposure additionally protected from Ers changes seen in the saline treated hyperoxia group, suggesting dose-dependent attenuation of BPD pathology in the lung parenchyma and airways. Of note, while lung mechanics did not show sexual dimorphism in these P21 studies, murine airway reactivity may additionally differ by sex in the post-pubescent animal at later timepoints [17].

Newborn mice are born with immature, saccular lungs [18]. This early exposure to both moderate hyperoxia and N6022 (P1-P5) was chosen to model GSNO reductase inhibition in human infants born prematurely in terms of similar saccular stage lung development. The assessments at the recovery timepoint (P21) were intended to model the effects of early GSNO reductase inhibition on the eventual lung morphology and mechanics in the alveolar stage. While the alveolar stage is estimated to end approximately at P28 in mice, in humans, the alveolar stage extends from 32 weeks gestational age into early school age [19].

These observed protections against neonatal hyperoxia may in part be attributed to the anti-inflammatory effects of GSNO [11,22]. Treatment with N6022 during moderate hyperoxia attenuated the inflammatory modulator TGF-β, which has been implicated in BPD pathophysiology [40,41], although further mechanistic studies and time points are warranted. Endogenous GSNO levels are kept in homeostasis through nitrosylation and denitrosylation events, including GSNO degradation by GSNO reductase. Indeed, the anti-inflammatory effects of GSNO reductase inhibition have been reported in other pulmonary diseases [38,42,43]. Of note, in balancing inflammation and nitrosative stress [44], GSNO reductase inhibition in this model did not result in increased protein damage in the form of nitrotyrosine. Newborn hyperoxia exposures in GSNO reductase knockout mice and wild-type mice supplemented with sodium nitrite have similarly not resulted in an overproduction of nitrotyrosine end-products [17,37].

While daily GSNO reductase inhibition provided potential protection in this hyperoxic model of BPD, it has several limitations. The hyperoxia murine model of BPD accounts for birth in the saccular stage of lung development and lung injury secondary to hyperoxia only; it does not fully account for the complex etiologies of human BPD (i.e., exposures to positive pressure ventilation, antenatal inflammation, perinatal and postnatal infection, respiratory instability, and comorbid complications such as necrotizing enterocolitis). In addition, in our current study, the lung pathology and respiratory mechanics were evaluated after 5 days of hyperoxia and recovery in room air to 3 weeks of age; however, adult outcomes still need further assessments, including evaluations of long-term emphysematous changes and pulmonary hypertension end-organ injury. N6022 was selected for these studies because of previous reports of drug pharmacokinetics, efficacy in animal lung models [25,26,36,38,39], and safety in human subjects with asthma [24]. Moderate neonatal hyperoxia was used because it induces an airway hyperreactivity phenotype [20,45,46,47]. However, alternative oxygen exposure paradigms or other complementary injury models [18,19,20] may have different outcomes. Similarly, additional investigation is needed to understand the effects of alternative GSNO reductase inhibitors [42,48], including alternate routes of inhibitor administration and alternative dosage schedules.

While the 14-day N6022 toxicology studies of Colagiovanni and colleagues were encouraging [26], the literature suggests that there may be downsides to lifelong GSNO reductase inhibition. It has been previously reported that GSNO reductase knockout mice have increased hypotension in response to endotoxin [49], elevated susceptibility to Klebsiella pneumonia [50], and increased risk for hepatocellular carcinoma [51]. Clearly, further work is needed to fully assess the safety profile, efficacy, and protective mechanisms of GSNO reductase inhibition in the neonatal period. Future directions include multiple time points, additional gene and protein expression patterns, angiogenesis, matrix remodeling, alveolar cell differentiation, myofibroblast activation, oxidative stress signaling, fibrosis, or other pathways implicated in the heterogeneous disease of BPD.

Previous work has shown that acute GSNO reductase inhibition can reverse airway hyperreactivity in a BPD mouse model, pointing to GSNO reductase inhibition as a potentially beneficial drug for the acute treatment of airway hyperreactivity in human BPD. In addition, we have now shown that early, daily treatment with a GSNO reductase inhibitor, such as N6022, given during the period of developing BPD has potentially protective effects, lasting beyond the acute treatment with N6022. Studying the effects of targeting the S-nitrosothiol pathway in human infants with BPD should be a priority, since a preventative drug has the potential to benefit the lives of many infants born prematurely.

## 5. Conclusions

These newborn mouse studies show that GSNO reductase inhibition during hyperoxia has the potential to diminish acute lung inflammation and prevent alveolar simplification, decreased airway tethering, and airway hyperreactivity in juvenile mice. GSNO-based therapies being developed for adult disease might be considered to treat or prevent BPD in at-risk newborns.

## 6. Patents

US Patents: USPTO 10,537,557 and 11,389,430 “Methods of treating respiratory disorders;” Case Western Reserve University

## Data Availability

The original contributions presented in this study are included in the article/Supplementary Material; further inquiries can be directed to the corresponding author.

## Supplementary Materials

The following supporting information can be downloaded at https://www.mdpi.com/article/10.3390/biomedicines14010015/s1. Figure S1: (a) TGF-β Chemiluminescence Peaks from Capillary-Based Automated Western; (b) TGF-β Uncropped Lane View from Capillary-Based Automated Western. Figure S2: (a) Nitrotyrosine Chemiluminescence Peaks from Capillary-Based Automated Western; (b) Nitrotyrosine Uncropped Lane View from Capillary-Based Automated Western. Figure S3: (a) IL-1β Chemiluminescence Peaks from Capillary-Based Automated Western; (b) IL-1β Uncropped Lane View from Capillary-Based Automated Western. Table S1: flexiVent Individual Data Points.

## Abbreviations

The following abbreviations are used in this manuscript:

BPD	Bronchopulmonary Dysplasia
FDA	Food and Drug Administration (United States of America)
FiO_2_	Fraction of inspired oxygen
GSNO	S-Nitrosoglutathione
IL-1β	Interleukin-1 beta
IQR	Interquartile Range
TGF-β	Transforming Growth Factor-beta

## Appendix A

### Appendix A.1. Antibody Optimization for Automated Simple Western

All antibodies used for protein quantification via automated Simple Western were purchased through Cell Signaling. Homogenized mouse lung was used to optimize dilution factor, lysate protein concentration, etc. (Table A1).

**Table A1:** Antibodies optimized for automated Western.

Antibody Target	Molecular Weight	Catalog	Diluent	Antibody Dilution	Lysate Concentration (µg/µL)
IL-1β	Mature: 17, precursor: 32	#12242	D2	1:25	1
Nitrotyrosine	Sum of all peaks	#CS9691	MF	1:50	1
TGF-β	Mature: 17, precursor: 59	#3711	MF	1:25	1

### Appendix A.3. Body Weights of Mice Treated with Saline or N6022

Body weights were recorded daily for weight-based dosing of saline vehicle or the GSNO reductase inhibitor, N6022. Body weights did not pass normality testing, a Kruskal–Wallis One-Way Analysis of Variance on Ranks did not detect a significant effect at P5 or P21 (*p* = 0.102 and *p* = 0.135, respectively); thus, post hoc testing was not performed to compare groups.

**Table A2:** Mouse P5 and P21 body weights.

Experimental Group	P5 Body Weight Median (IQR) (Grams)	P21 Body Weight Median (IQR) (Grams)
21% + Saline	3.3 (3.0, 3.6)	10.0 (9.4, 11.2)
60% + saline	3.3 (3.0, 3.5)	10.0 (8.4, 11.2)
60% + N6022 (0.1 mg/kg)	3.3 (3.1, 3.5)	11.0 (10.4, 12.3)
60% + N6022 (1 mg/kg)	3.5 (3.1, 3.8)	10.4 (10.0, 11.1)
60% + N6022 (10 mg/kg)	3.2 (3.1, 3.5)	11.2 (9.9, 12.2)
