# Supplementary material for: Pharmacologic Inhibition of S-Nitrosoglutathione Reductase Prevents Hyperoxic Alveolar and Airway Disease in Newborn Mice
Source: Biomedicines. 2025 Dec 20;14(1):15. doi: 10.3390/biomedicines14010015 (PMC12837470; doi:10.3390/biomedicines14010015)
Supplement: Supplementary file 1 [file biomedicines-14-00015-s001.zip › biomedicines-3991265-supplementary.pdf]

Supplementary Materials

Figure S1a. TGF-β Chemiluminescence Peaks from Capillary-Based Automated Western.

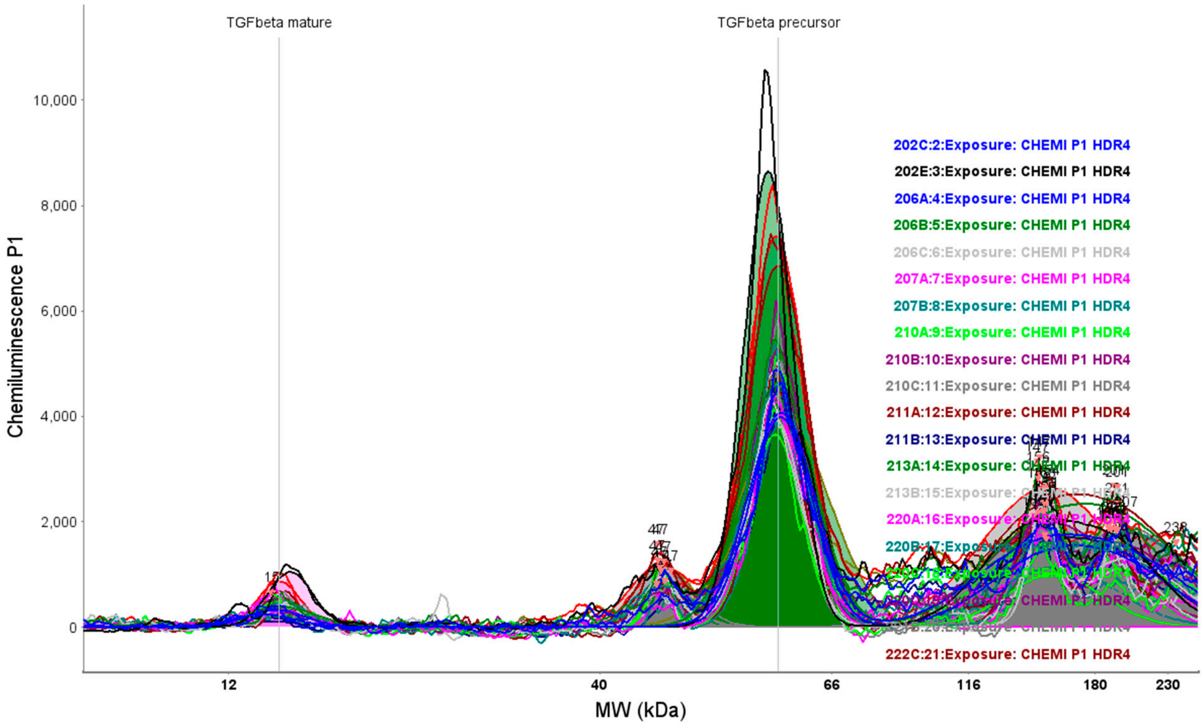

Figure S1b. TGF-β Uncropped Lane View from Capillary-Based Automated Western.

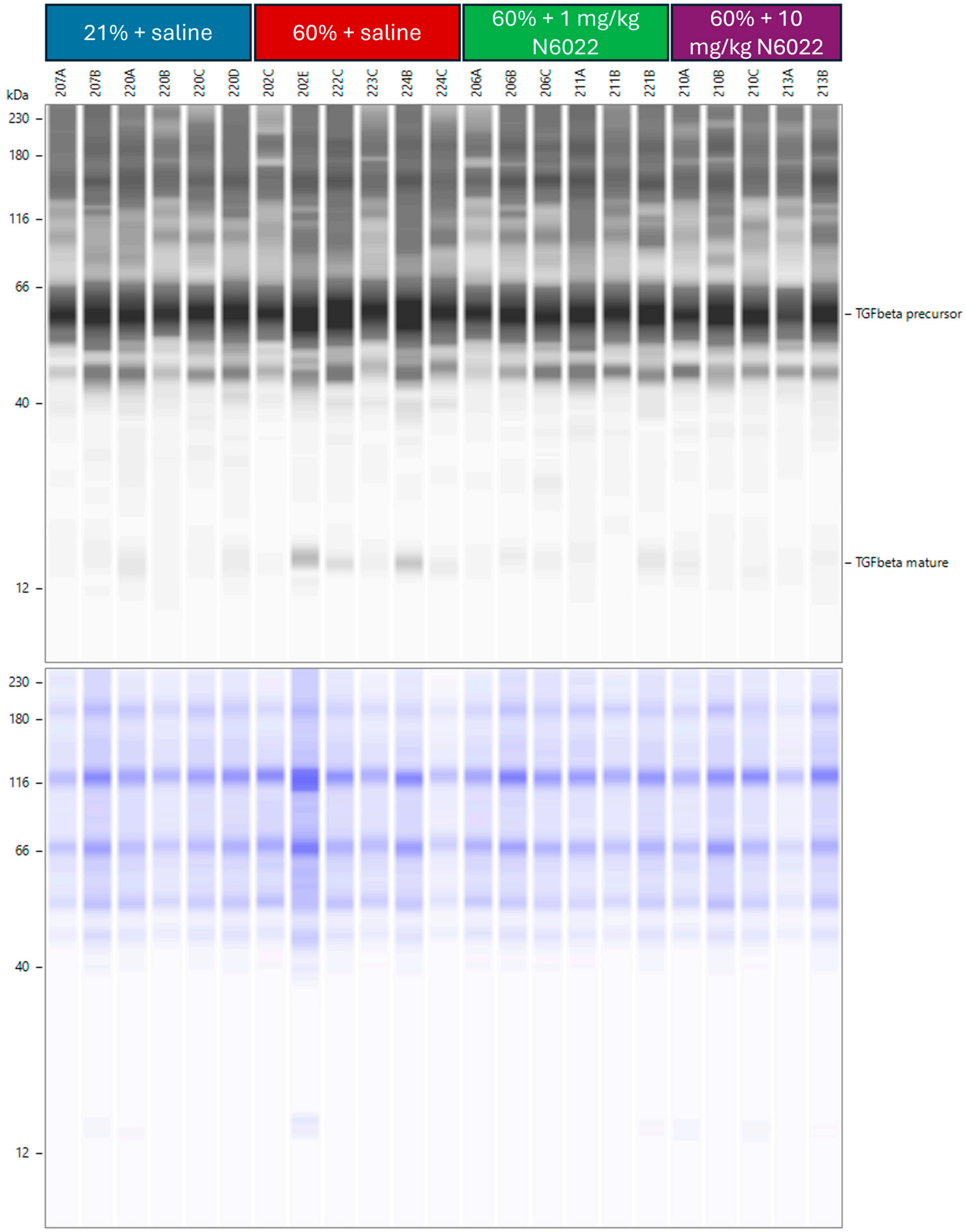

Figure S2a. Nitrotyrosine Chemiluminescence Peaks from Capillary-Based Automated Western.

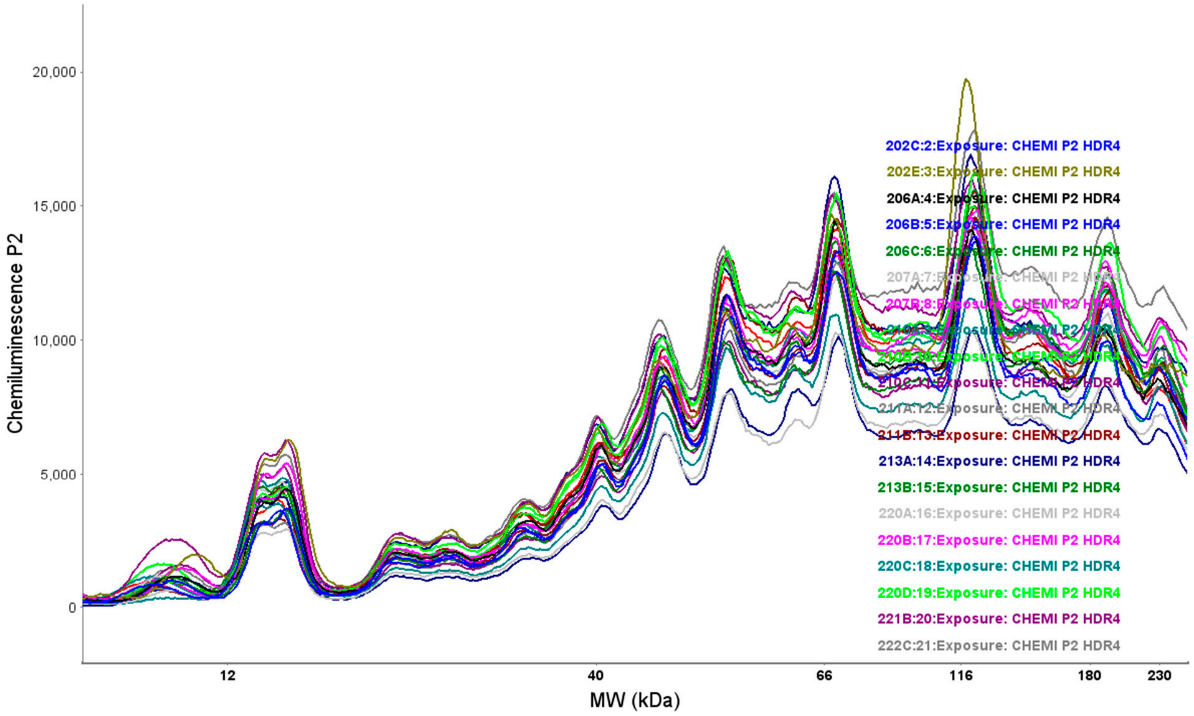

Figure S2b. Nitrotyrosine Uncropped Lane View from Capillary-Based Automated Western.

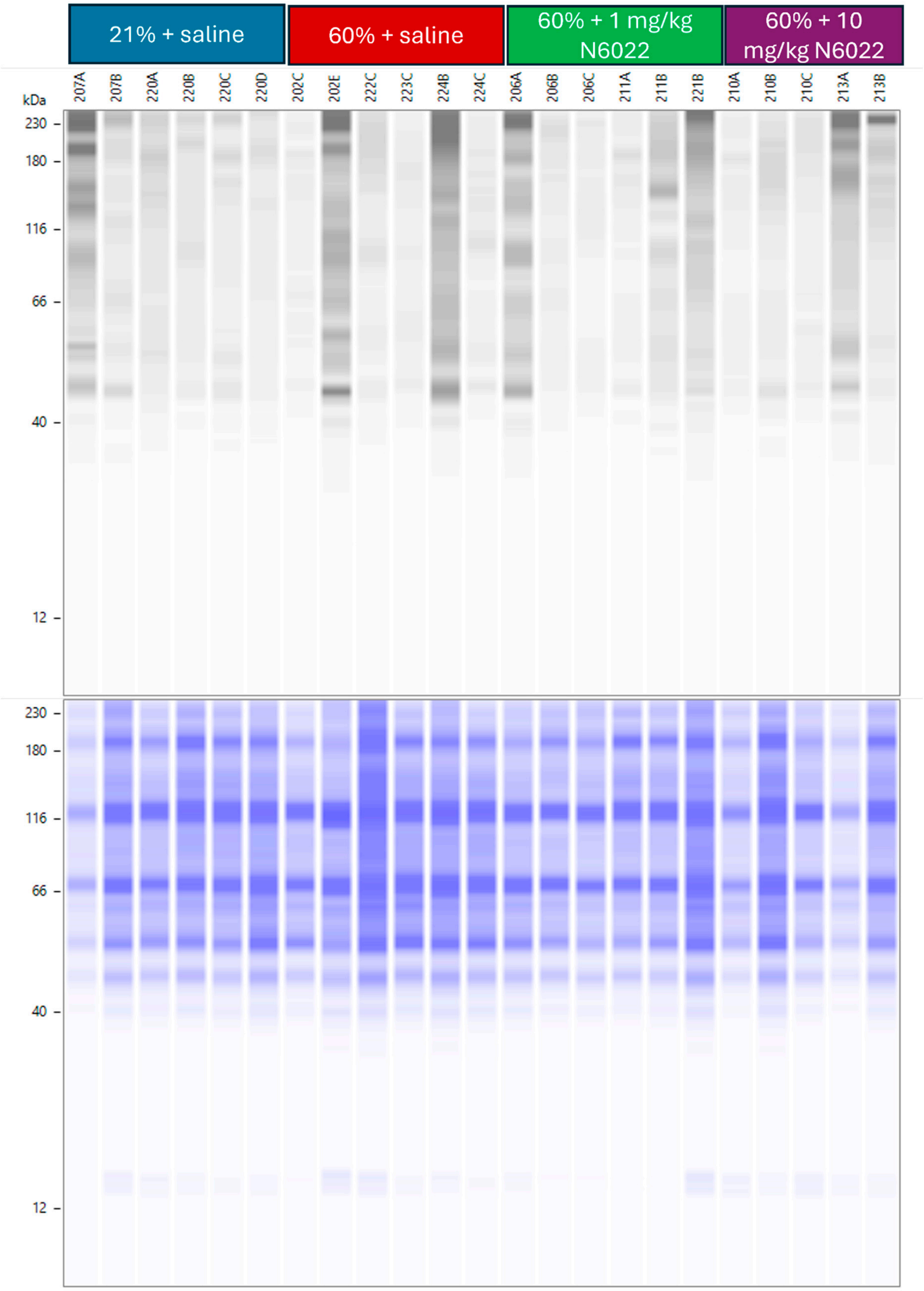

Figure S3a. IL-1β Chemiluminescence Peaks from Capillary-Based Automated Western.

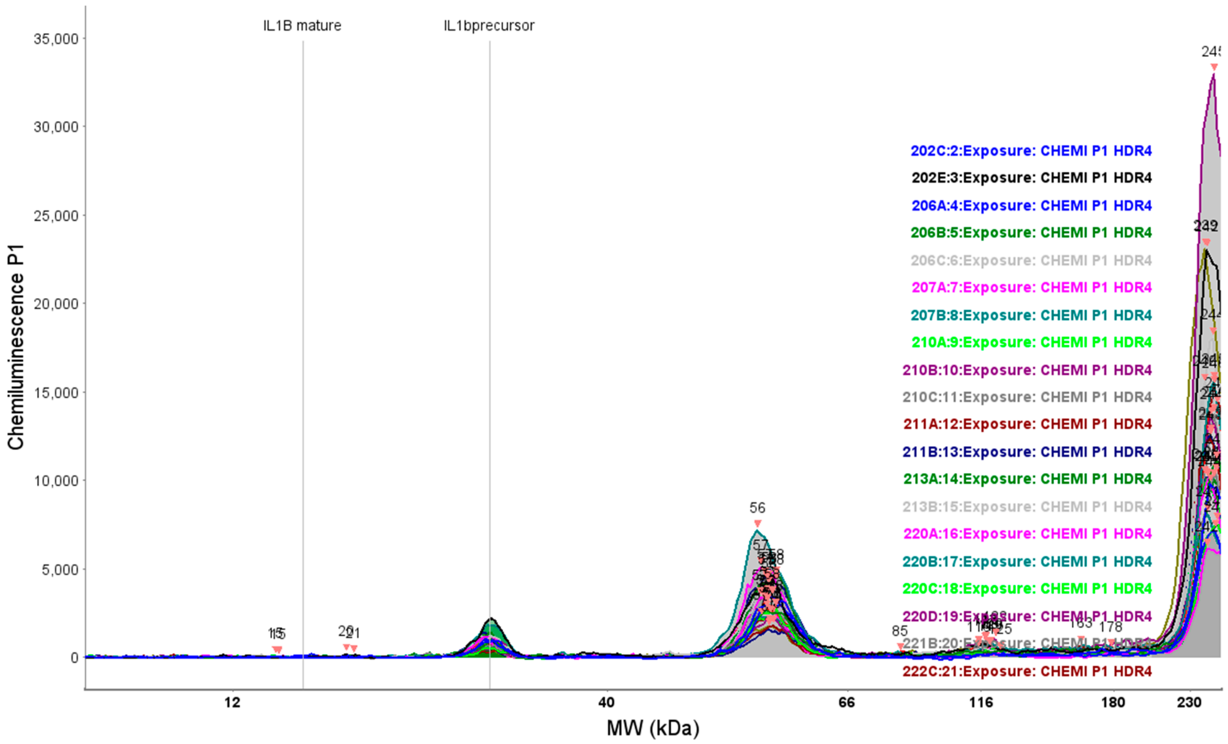

Figure S3b. IL-1β Uncropped Lane View from Capillary-Based Automated Western.

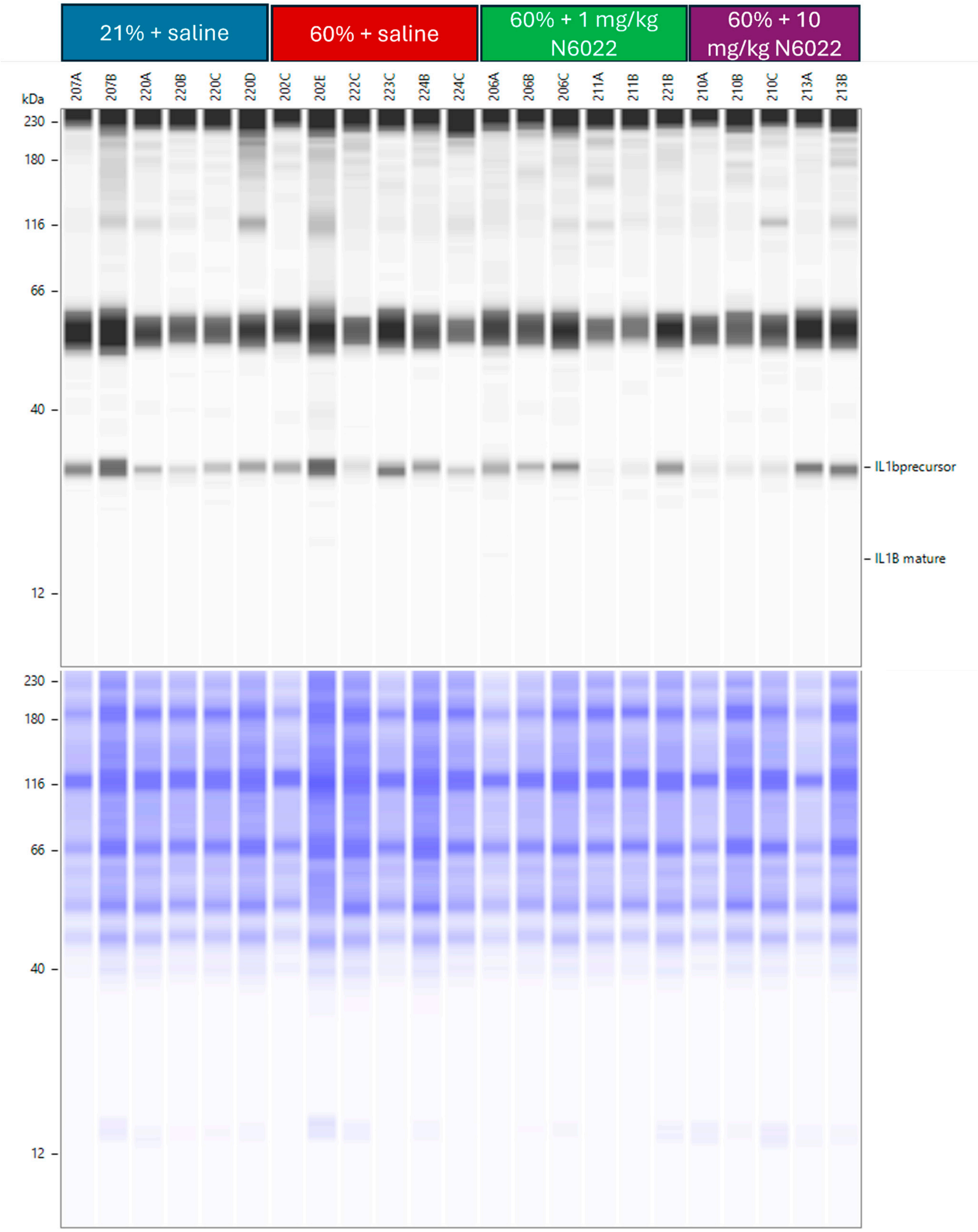

Table S1: flexiVent Individual Data Points

Animal ID#	Group	Mch Dose	Rrs	Crs	Ers	Sex (m=1)
1	1	1	1.2472	0.0151	66.2899	1
1	1	2	1.294	0.0142	70.6591	1
1	1	3	1.337	0.0136	73.3604	1
1	1	4	1.3687	0.013	76.7248	1
1	1	5	1.3988	0.0126	79.2301	1
1	1	6	1.4118	0.0127	78.7184	1
1	1	7	1.52	0.0123	81.641	1
1	1	8	1.7142	0.0118	84.9136	1
2	1	1	1.3632	0.0156	64.2031	1
2	1	2	1.3059	0.0158	63.3399	1
2	1	3	1.2848	0.0159	62.9681	1
2	1	4	1.4388	0.0162	61.6519	1
2	1	5	1.4834	0.0164	61.097	1
2	1	6	1.5341	0.0157	63.7548	1
2	1	7	1.573	0.0156	64.1675	1
2	1	8	2.0269	0.0147	67.856	1
3	1	1	1.3874	0.0163	61.3718	0
3	1	2	1.2639	0.0172	57.99	0
3	1	3	1.2003	0.0175	57.2212	0
3	1	4	1.1989	0.0179	55.8416	0
3	1	5	1.2386	0.0184	54.3603	0
3	1	6	1.3651	0.0187	53.517	0
3	1	7	1.5415	0.0186	53.8459	0
3	1	8	1.4973	0.0183	54.5771	0
4	1	1	1.3226	0.0143	70.1339	1
4	1	2	1.2464	0.0147	68.0044	1
4	1	3	1.2039	0.015	66.8294	1
4	1	4	1.2283	0.0148	67.7335	1
4	1	5	1.3109	0.0146	68.2948	1
4	1	6	1.4996	0.0141	71.0582	1
4	1	7	1.8186	0.0132	75.6227	1
4	1	8	2.0895	0.0125	79.839	1
5	1	1	1.4157	0.0132	75.5539	0
5	1	2	1.4294	0.0129	77.7872	0
5	1	3	1.3918	0.013	76.7982	0
5	1	4	1.4042	0.0132	75.8859	0
5	1	5	1.4657	0.013	76.7843	0
5	1	6	1.5532	0.0127	78.6981	0

5	1	7	1.8247	0.0121	82.9709	0
5	1	8	2.1093	0.0115	87.3095	0
6	1	1	1.1935	0.0201	49.8174	1
6	1	2	1.0627	0.0211	47.497	1
6	1	3	1.0206	0.0216	46.3717	1
6	1	4	1.0298	0.0218	45.8966	1
6	1	5	1.0796	0.0222	45.1309	1
6	1	6	1.203	0.0224	44.7375	1
6	1	7	1.486	0.022	45.4117	1
6	1	8	1.4747	0.0217	46.1884	1
7	1	1	1.1945	0.019	52.5469	0
7	1	2	1.1106	0.0197	50.89	0
7	1	3	1.0787	0.0204	49.1349	0
7	1	4	1.1128	0.0205	48.8071	0
7	1	5	1.1912	0.0205	48.907	0
7	1	6	1.3848	0.0203	49.2381	0
7	1	7	1.4015	0.0201	49.7814	0
7	1	8	1.7225	0.0185	55.1683	0
8	1	1	1.5527	0.0137	73.0137	0
8	1	2	1.3936	0.0143	70.1458	0
8	1	3	1.3125	0.0146	68.5802	0
8	1	4	1.3312	0.0147	68.2292	0
8	1	5	1.403	0.0147	67.9447	0
8	1	6	1.5398	0.0147	67.8489	0
8	1	7	1.7636	0.0148	67.7564	0
8	1	8	1.9392	0.0146	68.6119	0
9	2	1	1.742	0.0163	61.431	1
9	2	2	1.4066	0.0143	69.9429	1
9	2	3	1.2637	0.0141	71.0718	1
9	2	4	1.4172	0.014	71.6931	1
9	2	5	1.5574	0.014	71.4032	1
9	2	6	1.8295	0.0139	72.1215	1
9	2	7	2.6323	1.19E-02	87.9685	1
9	2	8	2.6257	8.29E-03	121.398	1
10	2	1	2.2296	9.13E-03	109.6009	1
10	2	2	2.6451	7.50E-03	135.2688	1
10	2	3	2.5674	7.68E-03	130.8394	1
10	2	4	2.4232	7.87E-03	127.1948	1
10	2	5	2.943	6.83E-03	146.6282	1
10	2	6	4.8277	5.96E-03	169.8183	1
10	2	7	5.0995	5.86E-03	172.1088	1

10	2	8	4.989	6.18E-03	162.0172	1
11	2	1	1.857	0.0148	67.7913	1
11	2	2	1.6355	0.0135	74.356	1
11	2	3	1.6334	0.0133	75.4489	1
11	2	4	1.6001	0.0133	75.1406	1
11	2	5	2.3153	0.0106	95.6977	1
11	2	6	3.2777	1.10E-02	93.4035	1
11	2	7	4.9945	5.71E-03	188.4795	1
11	2	8	7.2087	3.81E-03	262.7971	1
12	2	1	1.2244	0.0171	58.5968	1
12	2	2	1.3783	0.0152	65.7138	1
12	2	3	1.4212	0.0149	66.9443	1
12	2	4	1.4778	0.0147	68.0011	1
12	2	5	1.6233	0.0146	68.4847	1
12	2	6	2.5667	0.0145	68.9727	1
12	2	7	4.3019	0.0142	70.5732	1
12	2	8	5.5935	0.0149	67.1982	1
13	2	1	1.3305	0.0145	68.7667	0
13	2	2	1.4097	0.0135	74.3095	0
13	2	3	1.4563	0.0126	79.3791	0
13	2	4	1.4455	0.0124	80.7519	0
13	2	5	1.5086	0.0123	81.1707	0
13	2	6	2.7863	0.0124	80.6162	0
13	2	7	2.1915	0.0103	97.8116	0
13	2	8	3.0408	0.01	100.3951	0
14	2	1	1.361	0.0156	63.9254	0
14	2	2	1.4616	0.0145	69.2364	0
14	2	3	1.4638	0.0142	70.6628	0
14	2	4	1.4291	0.0141	70.9104	0
14	2	5	1.5239	0.0145	68.9139	0
14	2	6	2.1508	1.21E-02	97.8351	0
14	2	7	3.6474	6.25E-03	160.357	0
14	2	8	5.4263	6.61E-03	187.359	0
15	2	1	1.4358	0.0131	76.2748	0
15	2	2	1.4795	0.0126	79.5559	0
15	2	3	1.5114	0.0125	80.0242	0
15	2	4	1.511	0.0124	80.9434	0
15	2	5	1.4999	1.23E-02	81.3094	0
15	2	6	1.963	9.54E-03	107.713	0
15	2	7	2.1154	8.96E-03	112.1482	0
15	2	8	2.4754	8.86E-03	113.1509	0

16	2	1	1.5619	0.0124	80.9871	1
16	2	2	1.7201	0.0112	89.5589	1
16	2	3	1.7084	0.011	91.3327	1
16	2	4	1.6865	0.0109	91.554	1
16	2	5	1.696	0.0108	92.6821	1
16	2	6	1.8838	1.01E-02	98.9442	1
16	2	7	2.8088	8.36E-03	119.9136	1
16	2	8	3.152	8.42E-03	119.0153	1
17	2	1	1.048	0.0177	56.6243	0
17	2	2	1.1724	0.0153	65.4041	0
17	2	3	1.2032	0.0148	67.8036	0
17	2	4	1.3968	0.0128	77.9973	0
17	2	5	1.7244	0.0117	85.2865	0
17	2	6	1.9036	0.0114	88.0752	0
17	2	7	2.4883	0.011	90.6928	0
17	2	8	2.9766	0.0108	92.4214	0
18	2	1	1.1315	0.0168	59.6073	0
18	2	2	1.1703	0.0158	63.3203	0
18	2	3	1.1937	0.0155	64.6765	0
18	2	4	1.2092	0.015	66.7497	0
18	2	5	1.2351	0.0147	68.0931	0
18	2	6	1.2584	0.0147	68.3394	0
18	2	7	2.1162	0.0106	94.4741	0
18	2	8	2.9878	0.0104	97.6643	0
19	3	1	1.4542	0.0134	74.6328	1
19	3	2	1.5315	1.16E-02	85.9381	1
19	3	3	1.8365	9.73E-03	102.954	1
19	3	4	2.0583	8.77E-03	114.0853	1
19	3	5	2.1345	8.54E-03	117.1009	1
19	3	6	2.3553	8.13E-03	123.0314	1
19	3	7	2.903	7.57E-03	132.2018	1
19	3	8	3.3883	7.00E-03	142.9027	1
20	3	1	1.861	1.02E-02	98.3792	1
20	3	2	2.1992	8.12E-03	123.23	1
20	3	3	2.2131	7.92E-03	126.3433	1
20	3	4	2.2648	7.93E-03	126.1962	1
20	3	5	2.4585	7.35E-03	136.3194	1
20	3	6	3.2063	5.96E-03	171.6825	1
20	3	7	3.2896	5.98E-03	167.3306	1
20	3	8	3.767	5.17E-03	193.6063	1
21	3	1	1.3657	0.0164	60.8132	0

21	3	2	1.4693	0.0153	65.2941	0
21	3	3	1.6105	0.0145	68.878	0
21	3	4	1.3145	0.0136	73.3646	0
21	3	5	1.3577	0.0132	76.0143	0
21	3	6	1.8823	1.11E-02	90.7368	0
21	3	7	3.4397	9.43E-03	106.1393	0
21	3	8	3.2452	9.43E-03	106.2017	0
22	3	1	1.4658	0.0137	72.7788	0
22	3	2	1.5322	0.0126	79.4776	0
22	3	3	1.5732	0.0125	80.2081	0
22	3	4	1.5225	0.0118	84.7248	0
22	3	5	1.5394	0.0114	88.0559	0
22	3	6	1.5573	0.0114	87.552	0
22	3	7	1.7025	0.0109	91.6437	0
22	3	8	2.3353	0.0104	96.4794	0
23	3	1	1.3888	0.0133	75.3552	1
23	3	2	1.5366	0.0119	84.3127	1
23	3	3	1.4392	0.0123	81.1402	1
23	3	4	1.4735	1.21E-02	82.5858	1
23	3	5	1.9319	9.50E-03	105.3233	1
23	3	6	2.0073	9.55E-03	104.8288	1
23	3	7	2.4857	8.81E-03	113.6006	1
23	3	8	2.7572	8.79E-03	113.8531	1
24	3	1	1.2519	0.0153	65.2526	0
24	3	2	1.2971	0.0147	68.0539	0
24	3	3	1.2866	0.0152	66.0793	0
24	3	4	1.2675	0.0147	68.3258	0
24	3	5	1.2456	0.0145	68.7865	0
24	3	6	1.2737	0.0144	69.2833	0
24	3	7	1.4084	0.0139	72.1794	0
24	3	8	2.1335	0.0116	86.8643	0
25	3	1	1.2846	0.0137	73.0287	0
25	3	2	1.2403	0.0133	75.3366	0
25	3	3	1.2498	0.0136	73.6234	0
25	3	4	1.2274	0.0137	73.2979	0
25	3	5	1.2418	0.0136	73.4579	0
25	3	6	1.2892	0.0134	74.8725	0
25	3	7	1.4575	0.0129	77.6303	0
25	3	8	1.6904	0.0128	78.0775	0
26	3	1	1.029	0.0164	61.0835	0
26	3	2	1.1041	0.0152	65.9157	0

26	3	3	1.112	0.015	66.6972	0
26	3	4	1.0956	0.0149	67.3362	0
26	3	5	1.0797	0.0149	67.0041	0
26	3	6	1.0917	0.015	66.51	0
26	3	7	1.1981	0.0145	68.9825	0
26	3	8	1.445	0.0139	72.0951	0
27	4	1	1.2079	0.0169	59.119	1
27	4	2	1.153	0.0175	57.1359	1
27	4	3	1.2325	0.0163	61.3409	1
27	4	4	1.3041	0.0161	62.1499	1
27	4	5	1.4082	0.016	62.6079	1
27	4	6	1.556	0.0157	63.7969	1
27	4	7	1.6606	0.015	66.5842	1
27	4	8	2.1491	0.0135	74.243	1
28	4	1	1.0683	0.0165	60.5444	0
28	4	2	1.1716	0.0153	65.2897	0
28	4	3	1.2135	0.0147	68.1806	0
28	4	4	1.1919	0.0148	67.5829	0
28	4	5	1.1897	0.0148	67.7018	0
28	4	6	1.2398	0.0148	67.4187	0
28	4	7	1.4865	0.0143	69.9968	0
28	4	8	1.9292	0.0128	78.3983	0
29	4	1	1.1632	0.018	55.4374	1
29	4	2	1.2158	0.0166	60.3794	1
29	4	3	1.2759	0.0162	61.5976	1
29	4	4	1.3165	0.0161	62.349	1
29	4	5	1.3841	0.0156	64.0221	1
29	4	6	1.4631	0.015	66.6411	1
29	4	7	1.6557	0.0143	70.2059	1
29	4	8	1.9168	0.0138	72.3386	1
30	4	1	1.2405	0.0162	61.8666	0
30	4	2	1.2962	0.0155	64.6803	0
30	4	3	1.4024	0.0153	65.3573	0
30	4	4	1.2632	0.0152	65.8034	0
30	4	5	1.3107	0.015	66.8535	0
30	4	6	1.4062	0.0145	69.1842	0
30	4	7	1.585	0.0137	73.0312	0
30	4	8	2.3329	1.33E-02	75.3287	0
31	4	1	1.8068	9.87E-03	101.3604	0
31	4	2	1.9584	9.21E-03	108.8421	0
31	4	3	1.9251	9.07E-03	110.4205	0

31	4	4	1.9149	8.94E-03	111.9982	0
31	4	5	1.9052	8.88E-03	112.7759	0
31	4	6	1.9139	8.88E-03	112.6674	0
31	4	7	2.0108	8.76E-03	114.2437	0
31	4	8	2.48	7.78E-03	128.7278	0
32	4	1	1.2977	0.0152	65.7441	0
32	4	2	1.378	0.0149	67.3186	0
32	4	3	1.5174	0.0148	67.5123	0
32	4	4	1.7654	0.0149	66.9784	0
32	4	5	2.1479	0.0143	69.8274	0
32	4	6	2.5254	1.39E-02	72.0378	0
32	4	7	3.2438	9.95E-03	100.6437	0
32	4	8	4.4126	9.50E-03	105.4769	0

Table Legend: Group 1 (21%+Saline), Group 2 (60%+Saline), Group 3 (60%+1mg/kgN6022), Group 4 (60%+10mg/kgN6022). Methacholine (Mch) Dose 1 (baseline), Dose 2 (0 mg/mL), Dose 3 (6 mg/mL), Dose 4 (12.5 mg/mL), Dose 5 (25 mg/mL), Dose 6 (50 mg/mL), Dose 7 (100 mg/mL), Dose 8 (200 mg/mL). Respiratory system resistance (Rrs), Respiratory system compliance (Crs), Respiratory system elastance (Ers).
